# ARBOALVO: A Bayesian spatiotemporal learning and predictive model for dengue cases in the endemic Northeast city of Natal, Rio Grande do Norte, Brazil

**DOI:** 10.1371/journal.pntd.0012984

**Published:** 2025-04-29

**Authors:** Mariane Branco Alves, Rafael Santos Erbisti, Aline Araújo Nobre, Taynãna César Simões, Alessandre de Medeiros Tavares, Márcia Cristina Melo, Rodrigo Moreira Pedreira, Jan Pierre Martins de Araújo, Marilia Sá Carvalho, Nildimar Alves Honório

**Affiliations:** 1 Departamento de Métodos Estatísticos, Instituto de Matemática, Universidade Federal do Rio de Janeiro, Rio de Janeiro, Brazil; 2 Departamento de Estatística, Instituto de Matemática e Estatística, Universidade Federal Fluminense, Niterói, Brazil; 3 Programa de Computação Científica da Fiocruz, Fundação Oswaldo Cruz, Rio de Janeiro, Brazil; 4 Instituto René Rachou, Fundação Oswaldo Cruz, Belo Horizonte, Brazil; 5 Centro de Controle de Zoonoses, Secretaria Municipal de Saúde de Natal, Rio Grande do Norte, Brazil; 6 Laboratório das Interações Vírus-Hospedeiros, Instituto Oswaldo Cruz, Fundação Oswaldo Cruz, Rio de Janeiro, Brazil; 7 Núcleo Operacional Sentinela de Mosquitos Vetores–Nosmove/Fiocruz, Rio de Janeiro, Brazil; Kenya Agricultural and Livestock Research Organization, KENYA

## Abstract

**Background:**

Urban arbovirus transmission is spatially and temporally heterogeneous. Estimating the risk of dengue through statistical models that consider simultaneous variability in space and time provides more realistic estimates of transmission dynamics, facilitating the identification of priority areas for intervention focused on surveillance and control. These models also enable predictions to support timely interventions for arboviruses like dengue, chikungunya, and Zika.

**Methodology/principal findings:**

We analyzed dengue case reports by epidemiological week and neighborhood in Natal, RN from 2015 to 2018. Temporal conditional autoregressive models were fitted using the Integrated Nested Laplace Approximation method. The predictors included a set of entomological, climatic and sociosanitary indicators with temporal lags, along with structures of temporal and spatial dependence. Additionally, we used an offset term to represent the expected number of dengue cases per neighborhood at each epidemiological week, under the hypothesis of homogeneity in the occurrence of cases across the municipality. We forecasted dengue case counts for the subsequent four weeks, addressing both zero occurrences and fluctuations during non-zero periods. Weekly risk dynamics were visualized through predictive maps, enabling the timely identification of neighborhoods with high and persistent dengue risk, that is, areas consistently exhibiting a high number of dengue cases that remained concentrated in the same location for several weeks. The optimal model revealed a significant rise in dengue occurrence probability during the observation week, associated with increased cases in the previous week, the *Aedes* egg positivity index from the prior four weeks, and the mean daytime temperature 6–8 weeks earlier. Dengue risk also rose with a one-standard-deviation increase in the density of the impoverished population per occupied area and the mean *Aedes* egg density index from the preceding 3–5 weeks.

**Conclusions/significance:**

The proposed Bayesian space-time analysis can contribute to the operational control of dengue and *Aedes aegypti* by identifying priority areas and forecasting dengue cases for the next four weeks. It also quantifies the effects of entomological, sociosanitary, climatic and demographic indicators on both the likelihood of dengue occurrence and the intensity of outbreaks.

## Author summary

Urban arbovirus transmission exhibits spatial and temporal heterogeneity. Focusing on dengue, we employed statistical models that account for spatiotemporal variability to provide a realistic assessment of transmission dynamics. By using dengue case data from Natal, RN (2015–2018) and incorporating entomological, climatic, and sociosanitary indicators, we forecasted case counts for the following four weeks. Our analysis identified significant correlations between increased dengue risk and key factors: reported cases in the previous week, the *Aedes* egg positivity index, and mean daytime temperature from preceding weeks. High-risk neighborhoods with persistent dengue transmission were pinpointed, emphasizing areas for targeted interventions. This Bayesian space-time approach supports operational control efforts by identifying priority areas and clarifying how various factors influence dengue transmission. The findings have significant public health implications, enabling more precise, proactive strategies to mitigate the incidence and impact of urban arboviruses.

## Introduction

Emerging arboviruses such as dengue, Zika, and chikungunya pose one of the most significant global public health problems [1–4]. They are primarily transmitted by *Ae*. *aegypti* (Linnaeus, 1762), a vector well-established in tropical and subtropical regions [5,6]. Dengue has been endemic in Brazil for over 30 years, with successive epidemics [7–10]. This ongoing challenge was compounded by the emergence of chikungunya virus in 2014 [2] and Zika virus in 2015 [4,11]. According to the data publicized by the Brazilian Health Ministry, 2023 saw 1,658,816 dengue cases (81.69 cases per 10,000 inhabitants), 154,800 probable chikungunya cases (7.62 cases per 10,000), and 7,292 probable Zika cases (0.36 cases per 10,000).

In Brazil, the co-circulation of dengue, Zika and chikungunya intensifies public health challenges, contributing to significant morbidity, mortality and economic burden [2,10]. Continuous monitoring strategies are crucial, particularly given the territorial disparities of disease transmission. While arbovirus case counts vary across time and space, patterns of occurrence may exhibit similarities in neighboring areas and during proximate time periods. Among Brazilian areas affected by dengue epidemics, Natal, the capital of Rio Grande do Norte, has faced recurrent outbreaks since the arbovirus emerged in 1996 [12], with noteworthy epidemics recorded in 2008, 2016, and 2022. Located in Brazil’s Northeast region, Natal experiences high tourist activity, potentially contributing to the co-circulation of arboviruses and the dispersal of their vectors [13–19].

Current strategies for controlling these arboviruses involve targeting their primary vector, *Ae. aegypti*, but have yielded unsatisfactory results. The spatial heterogeneity of urban arbovirus transmission highlights the need for strategies that account for territorial complexity and differences, including the identification of priority areas—an approach considered promising for effectively targeted control actions [20,21]. Integrating entomological, epidemiological, and socio-environmental information specific to each territory is essential for the comprehensive management of urban arboviruses. To develop a more rational and tailored approach suitable for countries where dengue is endemic or epidemic, current control strategies should be reevaluated, beginning with an understanding of the demographic, environmental and social factors that drive dengue fever transmission dynamics [22,23].

Reliable data on intra-urban vector population estimates, trends in susceptible human populations, and virus entry timing are essential for understanding epidemic dynamics but are often lacking. In their absence, proxies such as entomological, climatic and sociosanitary indicators serve as indirect influences on urban arbovirus cases. Climatic factors, such as temperature, impact *Ae. aegypti* populations and viral dynamics [24]. Sociosanitary factors are expected to directly affect mosquito populations and indirectly increase infection risks due to urban conditions and high population densities [20,23].

This study pertains to the methodology portfolio developed by the ARBOALVO project to stratify priority intervention areas for urban arboviruses transmitted by *Ae. aegypti* [23,25]. The underlying hypothesis of this project is that the spatial distribution of cases is not homogeneous across territories and over time. To address this, Bayesian space-time models were applied, incorporating spatially and temporally structured random effects and accounting for dengue occurrences in neighboring areas and adjacent time periods. The model used was Conditional AutoRegressive (CAR), which adjusts for such effects in each neighborhood-epidemiological week combination. This approach allows assessing the impact of explanatory variables on the epidemiological risk of arbovirus transmission [26]. These effects were jointly estimated, considering various dimensions of the transmission process. The resulting relative risks of dengue occurrence were mapped, formally accounting for estimation uncertainty and identifying regions with persistent and/or heightened risks over time.

The spatial and temporal heterogeneity of transmission poses a challenge to understanding the risk of epidemics, and hinders the design of strategic control measures. Thus, the quantitative analysis of epidemiological dynamics in a spatiotemporal framework, as enabled by the model class employed, can provide valuable insights to support the development of more effective control strategies. These measures can include focused campaigns in areas and periods of persistently high risk and monitoring the emergence of new high-transmission zones. This study aimed to i) identify and quantify the effects of entomological, climatic, sociosanitary and demographic indicators as potential explanatory factors for dengue occurrence in Natal, RN; ii) predict the time series of dengue cases for each neighborhood up to four weeks ahead; and iii) forecast dengue cases stratified by the municipality’s neighborhoods, generating dynamic risk maps over time.

## Methods

### Ethics statement

This study was conducted within the framework of ARBOALVO program, approved by the research ethics committee of the Oswaldo Cruz Institute (CEP-IOC), under Protocol No. 51057015.5.0000.5537.

### Area and design of study

**Fig 1:**
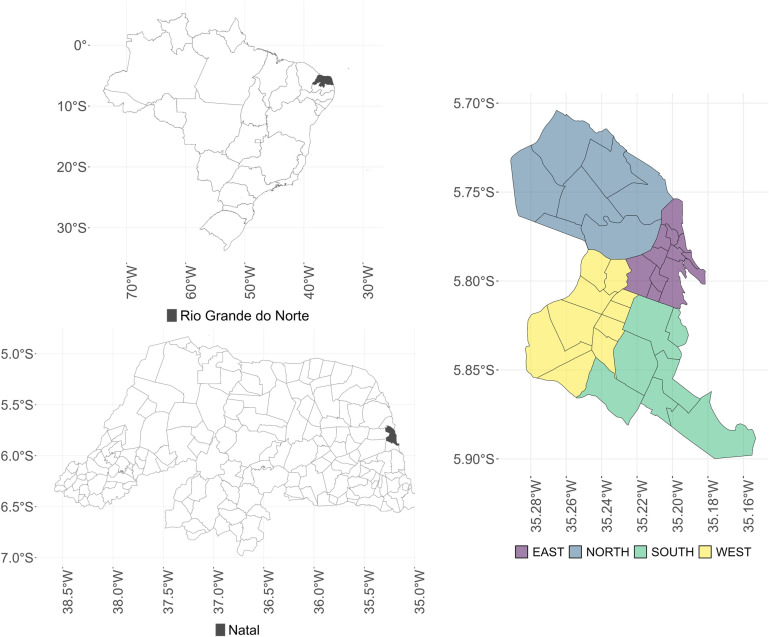
Map of the city of Natal by administrative regions. Source: Figure created by the authors. Shapefile of the neighborhoods in the city of Natal: https://www.natal.rn.gov.br/semurb/geoinformacoes. Shapefile of the municipalities in the state of Rio Grande do Norte and shapefile of the Brazilian states: https://www.ibge.gov.br/geociencias/organizacao-do-territorio/malhas-territoriais/15774-malhas.html.

This study employed an ecological analysis incorporating time-series and spatial heterogeneity methods to investigate factors influencing dengue occurrence in Natal, the capital of Rio Grande do Norte (RN) in Northeast Brazil. According to the Brazilian Institute of Geography and Statistics (IBGE), Natal has a population of approximately 896,708, ranking as the 20th most populous municipality in the country, with an area of 167 km² [27]. Located at 05°47’42“ S and 35°12’32” W at an altitude of 31 meters, Natal has a tropical climate with a dry summer, small temperature variation, and rainfall concentrated between March and July. The city is divided into 36 neighborhoods across four administrative regions, each characterized by distinct territorial, physical, demographic and infrastructural attributes (Fig 1). The eastern region comprises 12 neighborhoods, averaging 114,364 residents during the analysis period. The northern region includes 7 neighborhoods with a total population of 324,995. The western region consists of 10 neighborhoods with an average population of 226,734, while the southern region has 7 neighborhoods with an average of 170,364 residents. The population distribution by region and neighborhood for the analysis period is detailed in S1 Table. According to indicators such as the percentage of households with exclusive-use bathrooms and sewage disposal via rudimentary pits, trenches or natural water bodies; the percentage of households with open sewage in the vicinity; and the density of poor residents per occupied area, the highest sociosanitary deprivation was observed in neighborhoods in the northern and western regions [23,27]. Throughout this text, “occupied area” refers to the territorial space physically inhabited by people, defined as the ratio of the area occupied by anthropogenic structures to the total coverage area.

### Data source

The data used in this study are part of the ARBOALVO project—Methodological Proposal for Stratification of Risk Areas for Dengue, Chikungunya, and Zika in Brazilian Endemic Cities—funded by the Ministry of Health. The epidemiological data consist of reported dengue cases from 2015 to 2018, including laboratory-confirmed and suspected cases. These data were obtained from the Ministry of Health through the Information System for Notifiable Diseases (SINAN) of the SUS Informatics Department (DATASUS) and were subsequently aggregated by epidemiological week and neighborhood in Natal. Entomological data were sourced from the Municipal Health Department of Natal, consisting of weekly counts of eggs deposited by female *Ae. aegypti* mosquitoes in 400 oviposition traps (ovitraps). The ovitrap is a black plastic container (500 mL) with a Eucatex fiberboard paddle attached to its edge by a clip (details described in [28]). These traps were distributed in 36 neighborhoods from September 2015 to April 2018. The egg density index (EDI) was calculated by dividing the total number of *Aedes* eggs found on the paddles by the number of positive ovitraps. The egg positivity index (EPI) was calculated as the percentage of ovitraps positive for *Aedes* eggs, aggregated by neighborhood and epidemiological week. Sociosanitary and demographic data were obtained from the 2010 demographic census [29]. Climatic data, including precipitation and day and night surface temperatures, were obtained from two datasets: satellite data from the International Research Institute for Climate and Society (IRI) at Columbia University, and rainfall data from a station located in Natal, provided by the National Institute of Meteorology (INMET). The temporal window of the study, encompassing all available data, was defined by the span of the entomological data, which ranged from the 37th epidemiological week of 2015 to the 16th epidemiological week of 2018. Indicators derived from the databases at the neighborhood level of Natal are presented in S2 Table.

### Statistical analysis - preliminary stages to modeling dengue cases

#### Data imputation and scale compatibility of climate data.

Day and night temperature data gathered by the satellite were recorded every eight days, with some periods having missing data. Similarly, entomological data were incomplete in certain periods due to loss or other issues related to the ovitraps. To address these gaps, missing records of day and night temperatures, as well as *Aedes* egg density and positivity, were imputed using temporal conditional autoregressive models (temporal CAR) [30]. Climatic variables exhibit varying temporal and spatial scales across various data sources. Precipitation data from satellites were provided on a per-neighborhood and monthly basis, while daily rainfall data were obtained from a single station within the municipality. To ensure compatibility on the epidemiological weekly time scale, data from both sources were combined by multiplying the daily rainfall proportion from the municipal station by the monthly data for each neighborhood. This approach assumes a uniform rainfall regime across all neighborhoods and depends on data availability. For temperature data, which were provided by the satellite every eight days, a weighted average was calculated for each epidemiological week. The weights were based on the number of overlapping days between the observed epidemiological week and each eight-day satellite cycle. After this process, a comprehensive and compatible database containing temperature and rainfall data for all epidemiological weeks and neighborhoods was developed.

#### Analysis of the lagged impact of entomological and climatic indicators.

One of the initial steps in developing the spatiotemporal models proposed in this study was to evaluate the effects of entomological and climatic indicators at different time lags on dengue cases. For instance, it is unreasonable to assume that egg density observed in a specific epidemiological week would immediately influence dengue occurrence. Similarly, other climatic indicators do not immediately affect the outcomes. To address this, we adopted purely temporal models for the entire municipality to assess the lagged effects of these variables on dengue transmission. Let $Y_{t}$ denote the number of dengue cases in the municipality of Natal during week t, and $X_{t}$ represent a set of indicators (e.g., average rainfall volume, average egg density, proportion of positive ovitraps, and average daytime or nighttime temperature) at period t. A distributed lag structure [31,32] is assumed, which can be expressed as:


$$\begin{array}{c} Y_{t}\sim Poisson\left(\mu_{t}\right)\\ \log\left(\mu_{t}\right)=\zeta +\gamma_{0}X_{t}+\gamma_{1}X_{t-1}+\ldots +\gamma_{q}X_{t-q}. \end{array}$$ (1)

The proper estimation of the coefficients $\gamma_{0},\gamma_{1}\ldots ,\gamma_{q}$, would provide insight into the temporal propagation pattern of the impact of variations in the indicator X, observed *q* periods ago, on the response Yat the current time. However, from a statistical perspective, the estimation of these coefficients is hindered by the association present in indicator X across successive periods. One way to address this issue is to approximate the trajectory of the coefficients $\gamma_{j}$ (as a function of the lag order j) using a low-degree (d≪q) polynomial. The following reparameterization was applied:


$$\gamma_{j}=\sum_{k=0}^{d}\eta_{k}j^{k},j=0,1,2,\ldots ,q.$$ (2)

Rewriting the parameters $\gamma_{j}$ in (1), according to the approximation (2), results in an expression for the predictor in terms of d+1 parameters ($\eta_{0},\eta_{1,},\ldots ,\eta_{d}$):


$$\begin{array}{c} \log\left(\mu_{t}\right)=\eta_{0}S_{t0}+\eta_{1}S_{t1}+\ldots +\eta_{d}S_{td,}\\ S_{tk}=\sum_{j=0}^{q}j^{k}X_{t-j}.k=1,\ldots ,d. \end{array}$$ (3)

For smooth trajectories, a low-degree (d≪q) polynomial is sufficient to approximate the lag effects. Additionally, the new explanatory variables $S_{tj}$, obtained by aggregating the original indicators across their various lags, do not suffer from the collinearity problem typically observed in the original indicators. This approach resolves the instability in the estimation of the individual coefficients $\gamma_{j}$, leading to more stable and reliable parameter estimates. As a result, the estimation of the trajectory of the lagged impact curve of X on Y is improved, providing a more robust understanding of the temporal dynamics in the model [32].

Applications of the distributed-lag method in an epidemiological context have been explored in studies by Schwartz [32], Zanobetti et al. [33], and Xiang et al. [34]. In the present study, this method was employed to assess the influence horizon of indicators such as the egg density index (EDI), egg positivity index (EPI), rainfall volume, daytime temperature, and nighttime temperature on dengue case counts. The analysis was conducted using the dlnm package [35] in the statistical software R [36] to determine the appropriate number of time lags for each indicator included in the spatiotemporal models that consider the counts of dengue cases, in addition to entomological, climatic and sociosanitary variables.

#### Selection of Sociosanitary Indicators.

Building predictors based on combining several indicators can compromise the practical application of statistical models. Furthermore, uncertainties associated with estimated coefficients and adjusted values may be influenced by the inclusion of terms that do not effectively contribute to predictive capacity. Variable selection methods, which aim to reduce the dimensionality of the explanatory variable set, are valuable in this context. In this study, we applied the LASSO (Least Absolute Shrinkage and Selection Operator) regularization method [37], which imposes a constraint on model coefficients by shrinking them towards zero, effectively nullifying some coefficients. Therefore, this method serves as a variable selection approach. A preliminary analysis of various sociosanitary covariates was conducted using LASSO to identify those with the greatest ability to discriminate dengue cases. The sociosanitary indicators did not vary over time, since the source of the available data was the 2010 demographic census [29]. We adopted a simplifying assumption of temporal constancy of their impacts. Case counts were aggregated for the years 2016 and 2017, as well as over the entire analysis period. Thus, the LASSO procedure was applied to the accumulated dengue case counts at this stage to avoid the issue of excess zeros. The indicators identified as relevant in this preliminary analysis were then tested in the predictor of spatiotemporal CAR models in a subsequent stage. The R glmnet library [38] was used to implement the LASSO procedure, considering various neighborhoods as independent observations for each year. A Poisson distribution was assumed for the dengue occurrence counts. The initial selection of sociosanitary indicators as potential predictors was carried out according to a preliminary study using principal component analysis (PCA) [25]. At the conclusion of the LASSO procedure, the indicators marked with the symbol “*” in S2 Table were selected as candidates to compose the predictors for the models described in the following section.

### Mixed Bernoulli-Poisson spatiotemporal model

#### Proposed model.

In the more general spatiotemporal model adopted in this study, similar to the approach of Da Costa et al. [39], we assumed that the distribution of dengue cases could be represented by a mixture of two processes: a binary process (guided by a Bernoulli distribution) describing the absence or occurrence of cases, and a second process describing the counts of cases (guided by a Poisson distribution). For each combination of spatial unit i(i=1,…,N) and temporal unit t(t=1,…,T), $Y_{it}$ denotes a random variable representing the counts of cases, and we assumed that:


$$Y_{it}|\mu_{it}\sim \text{P}\left(\mu_{it}\right)$$

$$\log\left(\mu_{it}\right)=P_{it1}=log\left(E_{it}\right)+\alpha_{t}+\sum_{l=1}^{L}\beta_{l}^{y}x_{i,t,l}^{\left(y\right)}+\xi_{i,t}$$ (4)

$$\alpha_{t}=\alpha_{t-1}+\omega_{t},\omega_{t}\sim \text{N}(0,W)$$

$$\xi_{i,t}=\rho \xi_{i,t-1}+\upsilon_{i,t}$$

where $P(\mu_{it}\backslash )$ denotes the Poisson or zero-truncated Poisson distribution (truncated Poisson distribution at zero: $P\left(Y_{it}=0|\mu_{it}\right)=0$), with intensity $\mu_{it}$, which is linked by logarithmic transformation to a linear predictor $P_{it1}$described by L regressors, $x_{i,t}^{\left(y\right)}=(x_{i,t,1}^{\left(y\right)},\ldots ,x_{i,t,L}^{\left(y\right)})$, representing entomological, climatic and sociosanitary indicators recorded in neighborhoodi and time t. The vector $\beta^{y}=\left(\beta_{1}^{y},\cdots ,\beta_{L}^{y}\right)$ denotes the coefficients of the regressors in the predictor for counts of cases. For the relativization of case counts by the population living in the neighborhoods, *offset* terms ${log(E}_{it})$ were used, with $E_{it}$ denoting the expected number of cases per neighborhood at time t, assuming homogeneity in the distribution of cases throughout the municipality. The municipal rate of cases per inhabitant was multiplied, at each week, by the size of the resident population of each neighborhood: $E_{it}=\frac{{\text{reported cases}}_{t}}{{\text{population}}_{t}}\times {\text{population}}_{it}=\frac{\sum_{i=1}^{N}{\text{reported cases}}_{it}}{\sum_{i=1}^{N}{\text{population}}_{it}}\times {\text{population}}_{it}$. The projection for the inhabitants in each neighborhood was estimated based on the average annual growth rate between 2000 and 2010 derived from census data. We emphasize that in this study, the offset terms are primarily important due to the spatial variation of population sizes rather than temporal variation during the analysis period.

The linear predictor is also composed of a dynamic level $\alpha_{t}$ with a random walk temporal evolution, combined with a spatiotemporal structured error $\xi_{i,t}$, guided by the autoregressive coefficient ρ(|ρ|<1), allowing accommodation of possible overdispersion of the counts. The main purpose of including the effects $\xi_{i,t}$ is to formally account for temporal and spatial autocorrelation in case occurrences. These components reflect the notion that spatial and temporal dependencies within each neighborhood are linked to the space-time patterns of adjacent neighborhoods and previous weeks. This structure can be described by an intrinsic CAR specification [26] for the random effects $\upsilon_{i,t}$:


$$\upsilon_{i,t}|\upsilon_{-i,t},\tau \sim \text{N}\left(\frac{1}{n_{i}}\sum_{j\sim i}\upsilon_{j,t},\frac{1}{{\tau n}_{i}}\right)$$ (5)

where i~j≡j~i indicates that the neighborhoods i and j are adjacent and $n_{i}$ denotes the number of adjacent spatial units to neighborhood i. In this study, neighborhoods were considered adjacent if they shared borders. Thus, the spatial structure induced by $\upsilon_{i,t}$ considers an average of the process in neighborhood i, with increasing precision depending on the number of neighbors. Although quite intuitive, specification (5) results in an improper prior distribution for$\upsilon_{i,t}$, which can be corrected with the adoption of the specification


$$\upsilon_{i,t}|\upsilon_{-i,t},\tau ,\delta \sim \text{N}\left(\frac{1}{{\delta +n}_{i}}\sum_{j\sim i}\upsilon_{j,t},\frac{1}{\tau (\delta +n_{i})}\right)$$ (6)

where δ>0 is an additional parameter introduced on the main diagonal of the precision matrix. The specification (6) corresponds to the adoption of a precision matrix $Q=\{Q_{ij}\}$; $Q_{ij}=-\tau ;Q_{ii}=\tau \left(\delta +n_{i}\right).$ In this work, we adopted the specification proposed by Leroux et al. [30], where the precision matrix of the intrinsic CAR process is defined as a convex linear combination of the identity matrix (representing spatially independent effects) and the precision matrix (6): (1−λ)I+λQ. Thus, the parameter λ(0<λ<1) reflects the level of spatial structure in the data, with values close to one indicating strong spatial dependence and values near zero indicating spatial independence.

As previously mentioned, the general spatiotemporal model used in this study assumes a mixture of the Poisson or zero-truncated Poisson model for counts and a binary model for distinguishing null occurrences. Let $Z_{it}$ denote the binary variable such that


$$Z_{it}|\pi_{it}\sim \text{Bernoulli}(\pi_{it})$$ (7)

$$\text{log}\left(\frac{\pi_{it}}{1-\pi_{it}}\right)={P_{it2}=\beta }_{0}^{z}+\sum_{m=1}^{M}\beta_{m}^{z}x_{i,t,m}^{\left(z\right)}+\varphi \xi_{i,t}$$

where $1-\pi_{it}$ is the unobserved probability of belonging to the point mass component. Thus, the predictor $P_{it2}$ describes the occurrence or non-occurrence of dengue cases, structured in terms of parameters $\beta^{z}=\left(\beta_{0}^{z}{,\beta }_{1}^{z},\cdots ,\beta_{M}^{z}\right)$ for entomological, climatic and sociosanitary covariates, $x_{i,t}^{\left(z\right)}$, which may or may not overlap with the predictor $P_{it1}$ for case counts. The temporally and spatially structured random effect $\xi_{i,t}$ in (4) ensures smoothness in time and space for the binary process of case occurrence, scaled by a factor φ. From a Bayesian perspective, the model specification is completed with a prior distribution for the latent components. We assumed prior independence, expressed through the following distributions for the parametric vector Θ=(β,W,τ,δ,λ,φ,ρ), where $\beta =(\beta^{y},\beta^{z})$: $\beta \;\sim \text{MVN}(0,100000I_{\left(L+M\right)})$, where $I_{\left(L+M\right)}$ denotes the identity matrix of dimension (L+M)×(L+M), $W^{-1}\sim \text{Gamma}(0.0005,0.0005),$ τ~Gamma(0.0005,0.0005), δ~Gamma(1,1), $\text{logit}(\lambda \sim \text{N}\left(0,0.45^{-1}\right),$ φ~N(1,0.1), $\text{log}(\frac{1+\rho }{1-\rho }\sim \text{N}\left(0,0.15^{-1}\right)$.

The general spatiotemporal model is therefore composed of equations (4), (6) and (7) and can be represented by the following mixture form:


$$\left(1-\pi_{it}\right)I_{\left(Y_{it}=0\right)}+\pi_{it}p\left(y_{it}|\mu_{it}\right)=\left(1-\pi_{it}\right)\left({1-Z}_{it}\right)+\pi_{it}p\left(y_{it}|\mu_{it}\right),$$ (8)

where $I_{\left(Y_{it}=0\right)}$ is the indicator function of no occurrence of dengue cases; $p\left(y_{it}|\mu_{it}\right)$ denotes the Poisson or zero-truncated Poisson probability function (for a zero-inflated or hurdle formulation, respectively); and $\mu_{it}$ and $\pi_{it}$follow the predictive structures specified in equations (4), (6) and (7). The following cases of the general mixing structure in (8) were considered for comparison:

- *Poisson model*: $\pi_{it}=1$ and $p\left(y_{it}|\mu_{it}\right)$ is the usual non-truncated Poisson probability function, with predictive structure (4);
- *Hurdle Poisson model*: $p\left(y_{it}|\mu_{it}\right)$ is the zero-truncated Poisson probability function, with predictive structure (4), and $\pi_{it}=\pi$, which is equivalent to $\beta_{m}^{z}=\varphi =0,m=1,2,\ldots ,M.$ This implies that no predictive structure is assumed for the occurrence of zeros;
- *Zero-inflated Poisson model*: $p\left(y_{it}|\mu_{it}\right)$ is the usual non-truncated Poisson probability function, with predictive structure (4), and $\pi_{it}=\pi$, which is equivalent to $\beta_{m}^{z}=\varphi =0,m=1,2,\ldots ,M.$ This implies that no predictive structure is assumed for the occurrence of zeros;
- *Hurdle Logit-Poisson model*: $p(y_{it}|\mu_{it}\backslash )$is the zero-truncated Poisson probability function, with predictive structure (4). Additionally, a predictive structure for the mass point component was assumed, as described in Equation (7).

In addition to statistical criteria for the composition of predictors, the entomological and epidemiological plausibility of potentially relevant regressors for dengue transmission were also considered. The models described in this section were adjusted in the R software [36] with functions from the INLA package [40], which applies nested Laplace approximations to the posterior distribution.

#### Comparison of models and significance of variables.

We used the *Watanabe-Akaike information criterion* (WAIC) and *logarithm of the pseudo marginal likelihood* (LPML) [41] as statistical criteria for comparing the different models, which account for both the quality of the fit and the predictive capacity for unobserved periods and/or areas.

In classical or frequentist approaches, the significance of each regressor variable is typically assessed through a binary significance analysis to determine whether the unitary relative risk or odds ratio lies within a confidence interval. Since the models were fitted using a Bayesian approach, we instead examined the posterior estimated probability that the relative risk (or odds ratio) is greater (or smaller) than 1. If this probability significantly deviates from 0.5, we considered the effect of the corresponding regressor to be relevant.

#### Prediction of dengue cases over a k weeks horizon.

A municipality’s dengue risk levels can be evaluated through temporal maps depicting relative risks derived from spatiotemporal models. Bayesian methods enable these models to naturally generate predictive maps of the future spatial distribution of dengue cases. Such predictive distributions are essential since they can accurately represent future observations and align with the actual dynamics of the analyzed process. Let $y^{o}=(y_{1},\ldots ,y_{T}$) denote the vector of observed outcomes during the analysis period, with components $y_{t}={\left(y_{1t},\ldots ,y_{Nt}\right)}^{\prime }$, t=1,…,T, and assume an interest in generatingk week-ahead predictions for dengue occurrences $y^{f}=(y_{T+1},\ldots ,y_{T+k}$), where each component of the future value of observations has dimension N×1. The goal is to obtain the predictive distribution: $p\left(y^{f}|y^{o}\right)=\int p\left(y^{f}|\Theta \right)p(\Theta |y^{o})d\Theta ,$ where $p\left(y^{f}|\Theta \right)$ denotes the observational model for future observations, assumed to be identical to the model used for past observations, and $p(\Theta |y^{o})$ denotes the a posteriori distribution of the parametric vector, based on observations recorded up to time T. The predictive distributions were approximated using sampling methods, first by generating samples from the posterior distribution of the predictors $P_{it1}$ and $P_{it2}$ in (4) and (7), which were obtained using INLA for the Hurdle Poisson, Zero-inflated Poisson and Hurdle Logit-Poisson models. The points generated from the posterior samples were used to generate null observations or positive counts from structures (4) and (7), allowing us to obtain point predictions (given by the sample mean of the predictive distribution) and predictive intervals with a 95% credibility level, given by the 2.5th and 97.5th percentiles of the predictive distribution sample.

## Results

**Fig 2:**
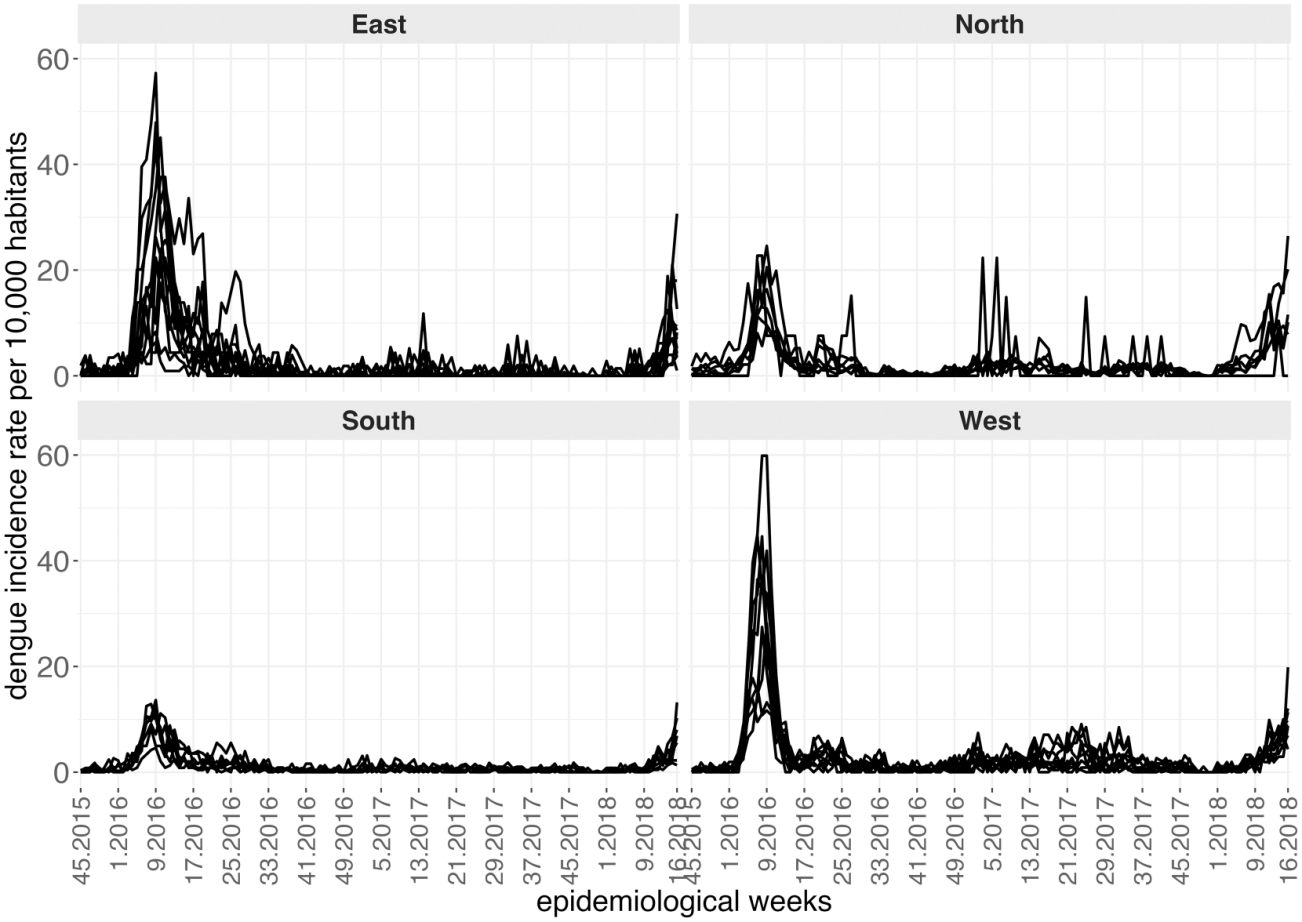
Time series of dengue cases, between the 45th epidemiological week of 2015 and the 16th week of 2018, for each neighborhood, according to administrative region, city of Natal, RN.

The analysis period spans from the 45th week of 2015 to the 16th week of 2018, during which 31,131 dengue cases were reported. In 2016, 14,907 cases were recorded, with an average monthly incidence rate of 14.65 cases per 10,000 inhabitants, while 5,334 cases were reported in 2017, corresponding to a monthly incidence of 5.19 per 10,000 inhabitants. Between September and December 2015, 622 cases were reported (monthly incidence of 1.85 per 10,000 inhabitants), and between January and April 2018, 10,268 cases were recorded (monthly incidence of 32.04 per 10,000 inhabitants). For the years analyzed, namely 2016 and 2017, the annual dengue incidence rates were 175.8 and 62.6 cases per 10,000 inhabitants, respectively. In April 2018, the cumulative annual incidence had already reached 128.2 cases per 10,000 inhabitants. Fig 2 illustrates the time series of dengue incidence rates per epidemiological week for each neighborhood in Natal, stratified by region. A peak in dengue cases was observed in March 2016 across all regions. The neighborhoods in the northern and western regions of the municipality had the highest number of cases, while those in the southern region did not exceed 50 weekly dengue cases throughout the study period.

**Fig 3:**
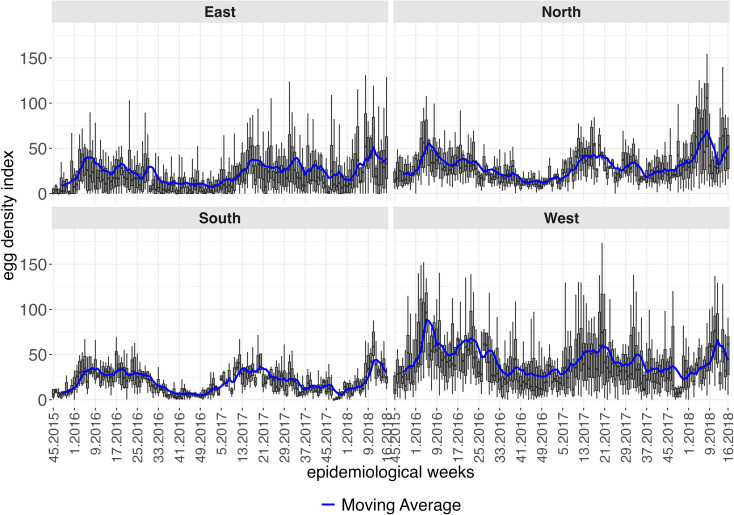
Boxplots of the egg density index across neighborhoods between the 45th epidemiological week of 2015 and 16th week of 2018, according to administrative region, city of Natal, RN. The solid lines represent the moving average of the egg density index in a temporal window of four epidemiological weeks.

Fig 3 presents the density of the *Aedes* egg index across neighborhoods, from the 45th epidemiological week of 2015 to the 16th week of 2018, with weekly boxplots grouped by administrative region. The solid lines in each panel represent the moving average of the indicator, calculated in a four-week temporal window. The southern region exhibited the lowest and most uniform levels of this indicator, aligned with the lowest frequency of dengue cases shown in Fig 2. Peaks in egg density were generally synchronous across all four regions and preceded the peaks in the epidemiological curves.

**Fig 4:**
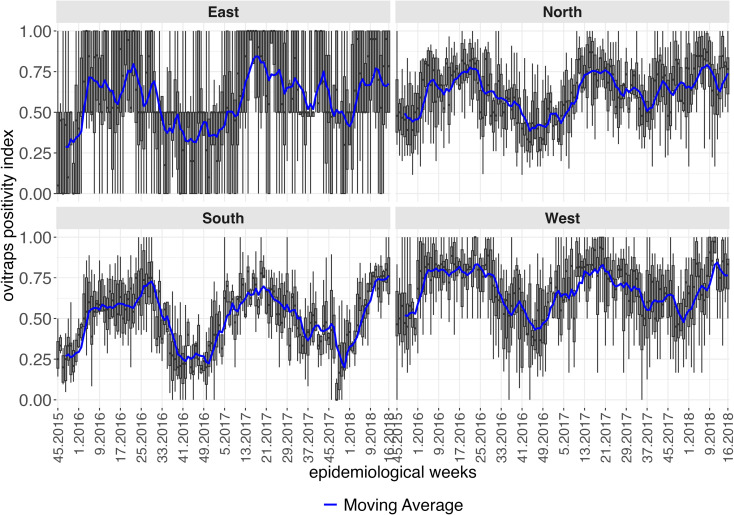
Boxplots of the egg positivity index across neighborhoods between the 45th epidemiological week of 2015 and 16th week of 2018, according to administrative region, city of Natal, RN. The solid lines represent the moving average of the egg positivity index in a temporal window of four epidemiological weeks.

For the *Aedes* egg positivity index, Fig 4 presents weekly boxplots indicating frequency of ovitrap saturation in the western, and especially the eastern regions. Periods were common where over 50% of ovitraps were positive in nearly all neighborhoods in the eastern, northern and western regions.

**Fig 5:**
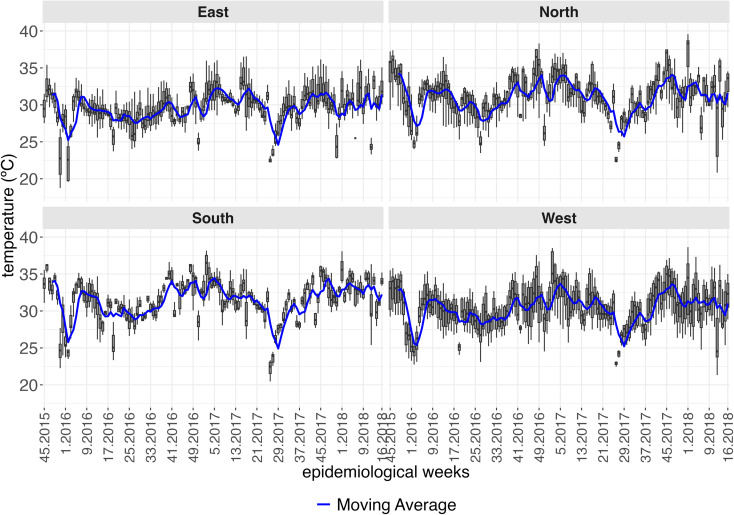
Boxplots of daytime temperature across neighborhoods between the 45th epidemiological week of 2015 and 16th week of 2018, according to administrative region, city of Natal, RN. The solid lines represent the moving average of daytime temperatures in a temporal window of four epidemiological weeks.

Fig 5 illustrates the evolution of weekly boxplots of average daytime temperatures across neighborhoods throughout the analysis period. A homogeneous pattern can be observed in the territory, characterized by consistently high temperatures, with peaks and declines occurring simultaneously in all four regions.

**Fig 6:**
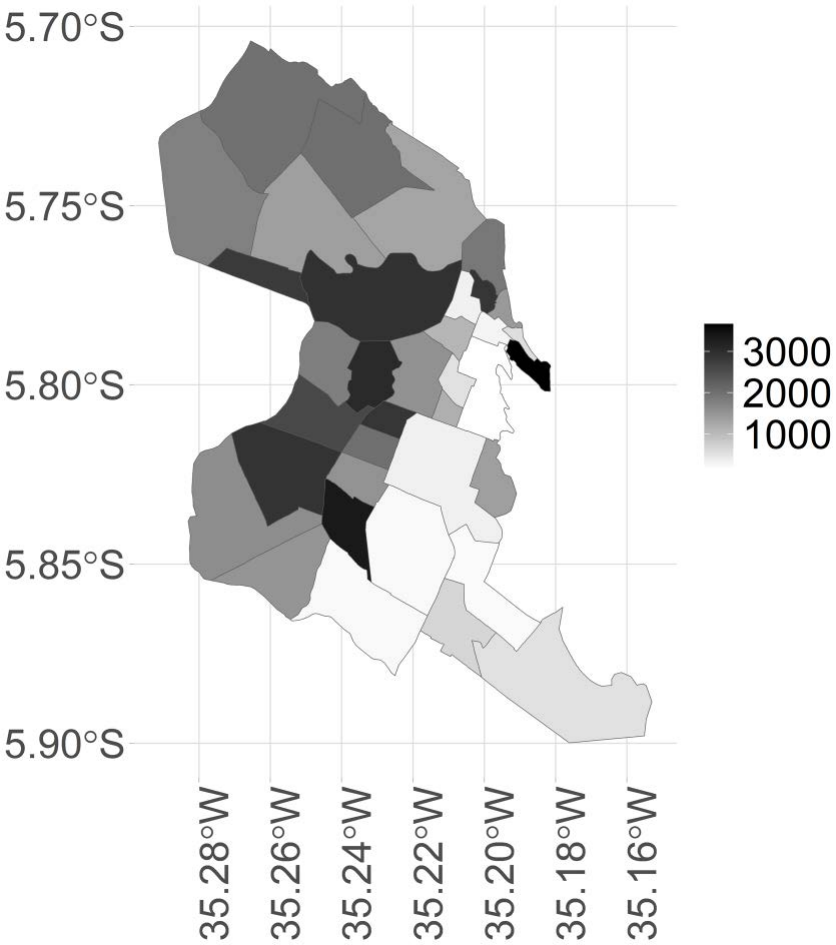
Stratification map by neighborhood of the poor population density indicator, by occupied area, city of Natal, RN. Source: Figure created by the authors. Shapefile of the neighborhoods in the city of Natal: https://www.natal.rn.gov.br/semurb/geoinformacoes

As shown in Fig 6, the northern and western regions, which recorded the highest number of cases during the analysis period (see Fig 2), also had the highest values of the poor population density indicator per occupied area. This indicator is defined as the number of households with a per capita monthly income of up to the monthly minimum wage per square kilometer of built area in a given spatial unit of analysis.

Polynomial lag models were applied with a maximum lag of q=12 weeks, using polynomials with degrees d=2,d=3, with the best results being obtained for d=3. These preliminary models helped guide the selection of lags to be applied to entomological and climatic indicators for explaining the number of dengue cases. The Poisson, Hurdle Poisson, Zero-inflated Poisson and Hurdle Logit-Poisson models were fitted with different predictive structures specified for the binary occurrence and count of cases. Sociosanitary indicators, selected using the LASSO procedure, were included as predictors in the CAR Bernoulli-Poisson models, and their significance was assessed within the predictive frameworks jointly established with other entomological and climatic indicators. The optimal composition for each predictor was selected from several predictive structures tested across the four models (Poisson, Hurdle Poisson, Zero-inflated Poisson and Hurdle Logit-Poisson). Table 1 presents the values obtained from the model selection criteria for the best fit of each model category. The adoption of the predictive structure for the Bernoulli portion of the model showed advantages over both the Hurdle Poisson and Zero-inflated Poisson models. The best results were clearly achieved with the Hurdle Logit-Poisson model, which combines a Bernoulli/Zero-truncated Poisson mixture and a predictive structure for both mixture components.

**Table 1:** WAIC and LPML for models’ comparison.

MODEL	WAIC	LPML
Poisson	15519.86	-7886.10
Hurdle Poisson	18048.40	-9205.85
Zero-Inflated Poisson	15517.78	-7880.08
Hurdle Logit-Poisson	**14294.14**	**-7274.86**

The Hurdle Logit-Poisson model, presented in Table 1, includes indicators of poor population density by occupied area (*vul14*) and the mean of the *Aedes* egg density indices (EDI) over the past three, four and five weeks. The regressors that make up the binary part of the model are dengue cases lagged by one week, egg positivity index (EPI) lagged by four weeks and average daytime temperatures lagged by six, seven and eight weeks. To obtain a standardized unit of variation for the indicators, we used increments of standard deviation to assess relative risks and odds ratios, as shown in Table 2.

**Table 2:** Summary measures associated with the posterior distribution of parameters comprising the predictive structure of the selected Hurdle Logit-Poisson model.

Binary portion of the model	*I*	Odds Ratio^a^		P^b^
		mean	CI 95%	
Intercept		9.76	(6.27; 15.27)	
Number of dengue cases (lag = 1 week)	14	9.39	(5.25; 15.97)	100.0%
EPI (lag = 4 weeks)	0.29	1.20	(1.09; 1.33)	99.9%
Daytime temperature (lags = 6,7,8 weeks)	2.5	1.37	(1.20; 1.57)	100.0%
**Poisson portion of the model**	*I*	**Relative Risk** ^a^		**P** ^b^
		**mean**	**CI 95%**	
Intercept		0.59	(0.37; 0.94)	
EDI (lags = 3,4,5 weeks)	26	1.03	(0.99; 1.07)	90.9%
Poor population density	1013	1.20	(1.13; 1.28)	100.0%

^a^Associated with increment *I*.

^b^P: Posterior probability of relative risk/odds ratio greater than 1, associated with increment *I* on the regressor.

The results presented in Table 2 indicate that for each increment of one standard deviation in the density of poor people per occupied area (1,013 poor per km² of occupied area), the risk of dengue increased by 20%. For each standard deviation increment in the egg density index (averaging 26 *Aedes* eggs per ovitrap per neighborhood per week), the estimated risk of dengue rose by 3%. The estimated posterior probability of a significant positive impact was 90.9%. Hence, with high probability, increases in EDI levels were associated with an increase in the number of expected cases. Regarding the occurrence of dengue cases (binary part of the model), it was estimated that for each standard deviation increase (14 cases of dengue) in the number of cases from the previous week, the odds of having cases in the current week increased 8.39 times. Additionally, for each standard deviation increase in the proportion of positive ovitraps (29% positive ovitraps on average, per neighborhood per week) from four weeks prior, the odds of cases in the current week increased by 20%. For each standard deviation increase in the average daytime temperature (2.5 ºC) from six to eight weeks ago, the odds of cases in the current week were expected to rise by 37%.

**Fig 7:**
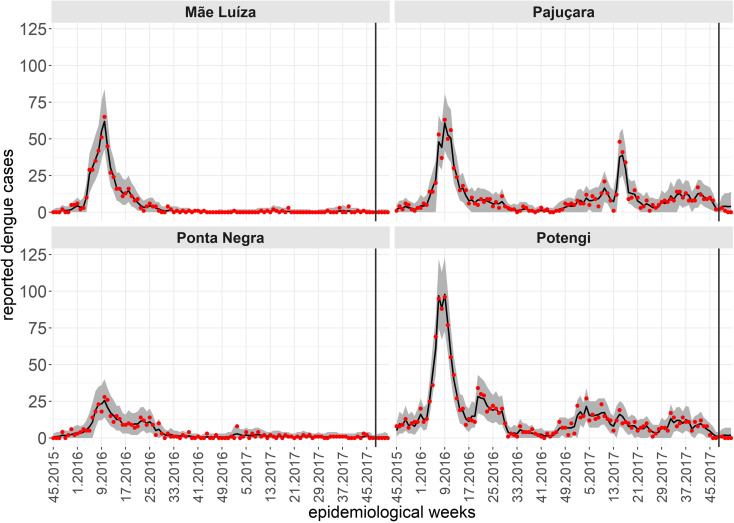
Fit and prediction of the average number of dengue cases for the selected neighborhoods in period$t_{1}$, which starts at the 45th epidemiological week of 2015. Prediction for the epidemiological weeks of 2017, city of Natal, RN. Solid line: Point adjustment/forecast. Gray region: Credibility interval at the 95% credibility level for the adjustment /forecast. Red dots: Reported dengue case counts.

**Fig 8:**
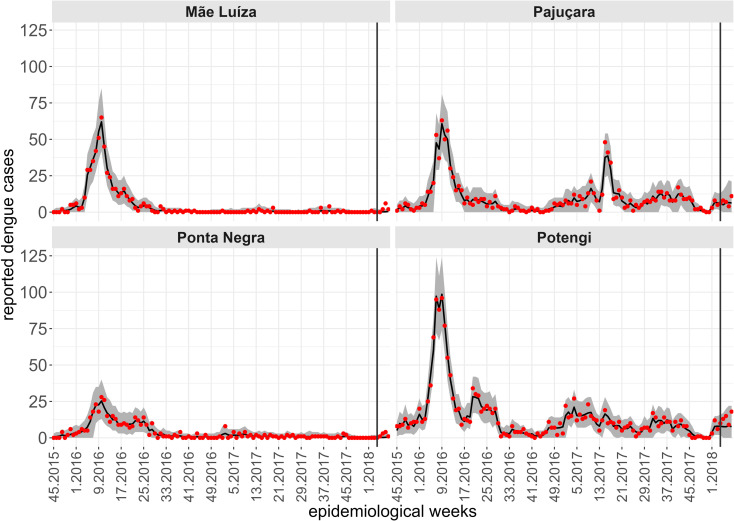
Fit and prediction of the average number of dengue cases for the selected neighborhoods in period$t_{2}$, which starts in the 45th epidemiological week of 2015. Prediction for the 5th to 8th epidemiological weeks of 2018, city of Natal, RN. Solid line: Point adjustment/forecast. Gray region: Credibility interval at the 95% credibility level for the adjustment /forecast. Red dots: Reported dengue case counts.

**Fig 9:**
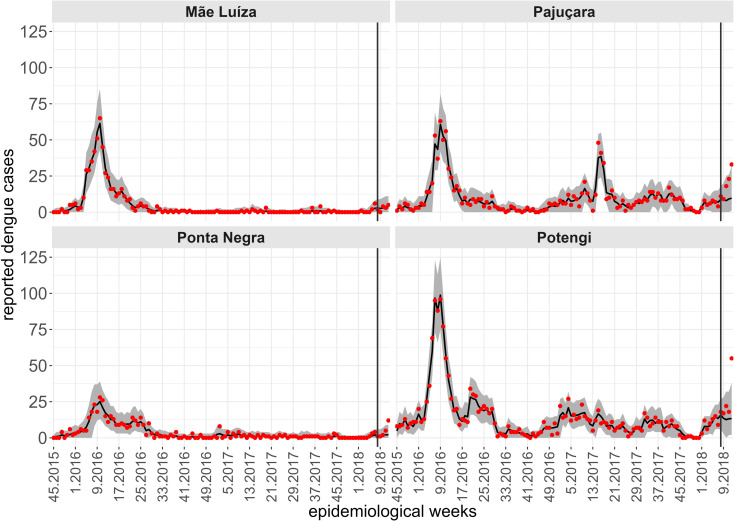
Fit and prediction of the average number of dengue cases for the selected neighborhoods in period $t_{3}$, which starts in the 45th epidemiological week of 2015. Prediction for the 9th to 12th epidemiological weeks of 2018, city of Natal, RN. Solid line: Point adjustment/forecast. Gray region: Credibility interval at the 95% credibility level for the adjustment /forecast. Red dots: Reported dengue case counts.

**Fig 10:**
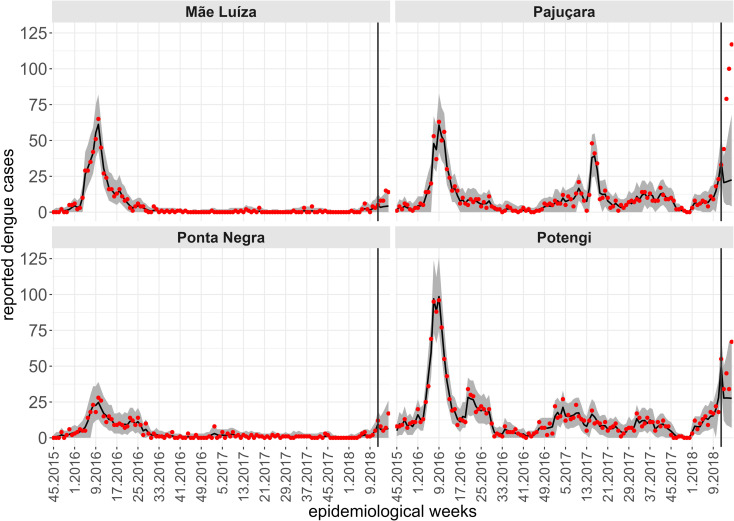
Fit and prediction of the average number of dengue cases for the selected neighborhoods in period $t_{4}$, which starts in the 45th epidemiological week of 2015. Prediction for the 13th to 16th epidemiological weeks of 2018, city of Natal, RN. Solid line: Point adjustment/forecast. Gray region: Credibility interval at the 95% credibility level for the adjustment /forecast. Red dots: Reported dengue case counts.

**Fig 11:**
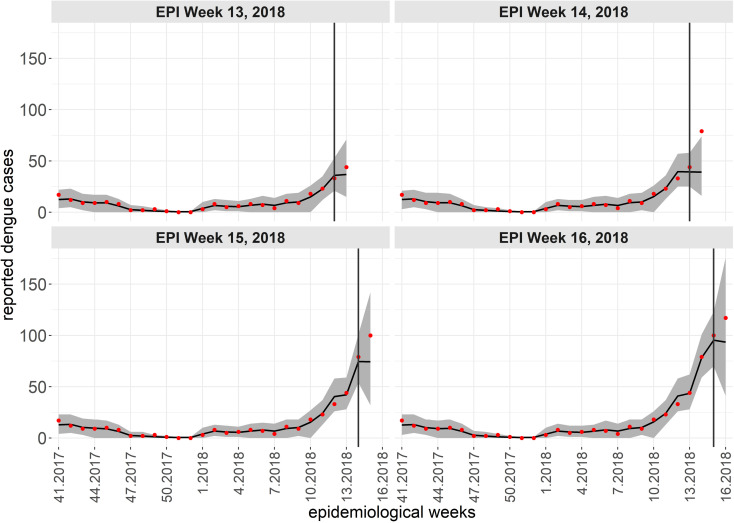
Fit and prediction of the average number of dengue cases for the Pajuçara neighborhood in period $t_{4}$, which starts at the 45th epidemiological week of 2015. Forecasts one week ahead, in the period between the 13th and 16th epidemiological weeks of 2018, city of Natal, RN. Solid line: Point adjustment/forecast. Gray region: Credibility interval at the 95% credibility level for the adjustment /forecast. Red dots: Reported dengue case counts.

While a single spatiotemporal model provides results for all neighborhoods of the territory, to illustrate the predictive performance of the Hurdle Logit-Poisson model, we also present the results for four neighborhoods (Mãe Luíza, Pajuçara, Ponta Negra, and Potengi), exhibiting distinct epidemiological processes. To assess the model’s predictive quality, we fitted four temporal windows, starting from the first data point available (the 45th epidemiological week of 2015). In each scenario, four-week forecasts were generated for periods: $t_{2}$, the 49th to 52nd epidemiological weeks of 2017; $t_{2}$, the 5th to 8th epidemiological weeks of 2018; $t_{2}$, the 9th to 12th epidemiological weeks of 2018; and $t_{2}$, the 13th to 16th epidemiological weeks of 2018. The forecasts four steps ahead are conditional on the lagged values of the entomological and climatic indicators, except for the number of cases lagged by one week. For this variable, we assumed a constant and identical value corresponding to the average of the last four weeks of observations. Figs 7-10 show the results of the Hurdle Logit-Poisson model for the four selected neighborhoods and forecasts four weeks ahead for horizons $t_{2}$, $t_{2}$, $t_{2}$ and $t_{2}$, respectively. The vertical lines in Figs 7–11- mark the moment of interruption of the adjustment and start of the forecasting period.

As expected, the credibility intervals for the predictions widened with the predictive horizon and successfully captured four weeks of future dengue case counts, except during periods of sudden and rapid growth, as demonstrated by the forecast for the Pajuçara neighborhood, shown in Fig 10.

Fig 11 shows the updated revised forecasts for the Pajuçara neighborhood in the $t_{4}$ period, using a one-week predictive horizon and accommodating new case data each week to forecast the following week. The model quickly adapted to the accelerated growth of dengue cases, providing satisfactory forecasts when updated frequently. Moreover, the predictive intervals adapted to the increasing magnitude of the number of dengue cases. Forecasting should be evaluated not only based on the point estimates, but also by considering the associated uncertainty.

**Fig 12:**
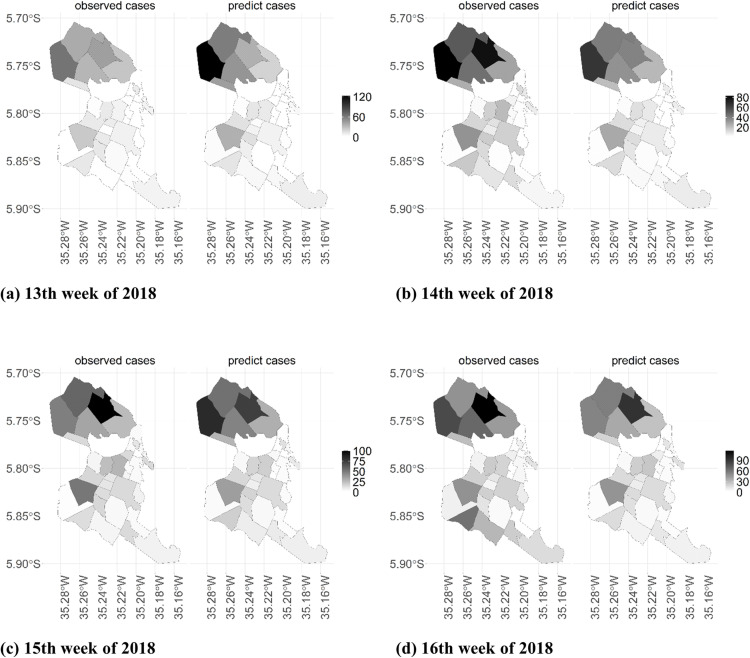
Stratification maps of observed and predicted dengue case counts for four epidemiological weeks ahead (between the 13th and 16th epidemiological weeks of 2018), city of Natal, RN.

Fig 12 displays maps of the stratification of dengue cases across the entire municipality of Natal based on predictions from the Hurdle Logit-Poisson model in the $t_{4}$ predictive window. These maps were generated using a one-week forecast of dengue case counts. Notably, the Hurdle Logit-Poisson model demonstrated an enhanced ability to predict areas with higher case intensity. Throughout all evaluated weeks, the high-risk areas identified by the forecasts were subsequently validated by dengue case counts one week later. Additionally, the model effectively distinguished regions within the city with low or no dengue cases.

## Discussion

Dengue is the most prevalent mosquito-borne viral disease worldwide [42]. People from tropical and subtropical countries, including Brazil, can be infected with one or more of the four serotypes [3,43]. Statistical modeling estimates suggest that dengue causes approximately 390 million infections globally each year, including 96 million cases of classical symptomatic dengue and 20,000 deaths [44].

Dengue epidemics in Brazil have persisted over the years, with significant morbidity and mortality burdens on public health [10]. Therefore, it is of utmost importance to propose different strategies for the routine monitoring of this disease, considering the disparities in transmission across the country. Although dengue case counts exhibit heterogeneity in both time and space, it is reasonable to expect neighboring areas and close time points to be similar. To better understand the patterns in the occurrence of dengue in the municipality of Natal, we identified and quantified the effects of entomological indicators (oviposition indices), climatic factors (daytime and nighttime temperature), and sociosanitary and demographic indicators as potential explanatory factors for the occurrence of this arbovirus.

Our findings revealed that the largest effects of each indicator on the number of reported dengue cases in the municipality of Natal were as follows: i) the egg density index had the greatest impact at a lag of four epidemiological weeks, with notable effects also observed in the third and fifth lagged weeks; ii) likewise, the egg positivity index had its strongest effect at lag four; and iii) the daytime temperature had a significant influence at lags of six, seven and eight epidemiological weeks. We conducted a single fit of a polynomial lag model [32,35,52] capable of integrating the temporal trajectory of these effects along with the number of periods until the peak of the lagged effect and its subsequent decline, until it no longer had an impact. From a temporal perspective, it is crucial to note that the impact of variations in entomological and climatic indicators on the occurrence of dengue cases is not immediate. Some studies have sought to describe the temporal lag of the effects between predictor variables and the response variable by fitting various models based on individual or grouped lags of the predictors, which naturally poses limitations, as discussed by Sanchez-Gendriz et al. [17].

The seasonal dynamics of dengue transmission has been linked to the delayed effects of climate and entomological indices on vector population dynamics. In Brazil, one study showed that mean weekly temperatures above 22-24 °C were strongly associated with high *Ae. aegypti* populations, increasing the risk of dengue transmission. Both temperature and rainfall were significantly correlated with *Ae. aegypti* indices at a short (1 week) time lag [45]. In an epidemiological study of dengue occurrence in the municipality of Cambé, in the northern part of the Brazilian state of Paraná, temperature and the number of patients positive for dengue showed a positive cross-correlation with a lag interval of two months (eight weeks) [46]. In India, significant effects of temperatures between 26 and 28 °C on the relative risk of dengue incidence were estimated, with peak effects occurring around 5 weeks and persisting for up to 10 weeks [47].

Considering dengue cases in Natal, Brazil, Sanchez-Gendriz et al. [17] employed cross-correlation to estimate the temporal lag in the impact of the ovitrap density index on dengue incidence, finding a lag of 4 weeks. Betanzos et al. [48] observed a significant effect between ovitrap monitoring indicators and dengue incidence in Mexico, with a better fit achieved when considering the lagged effects of egg counts on case incidence. The authors obtained improved predictive results by incorporating egg counts with a lag of three weeks during the dry season and four weeks during the wet season, emphasizing that recognizing these lags enhances the predictive utility of indicators derived from ovitrap monitoring. The adoption of a single distributed lag structure for the entire territory of Natal is a limitation of our work, since these effects may vary over time and across spatial units. Estimating the spatiotemporal evolution of covariate effects propagation, as well as jointly modeling these effects, is part of our research agenda.

In our study, sociosanitary indicators did not exhibit temporal variation, since they were observed only during the census year. The application of a LASSO procedure [37] allowed the selection of a subset of sociosanitary indicators capable of explaining the spatial dynamics of dengue cases. Among the selected indicators were the density of impoverished individuals per occupied area and the percentage of households without access to urban sanitation services. Cabrera et al. [49] discussed how a low gross domestic product (GDP) per capita and high population density are potential indicators of dengue severity. Lowe et al. [50] explored the exposure-lag-response associations between hydrometeorological extremes and dengue risk in the months leading up to an outbreak using a Bayesian spatiotemporal formulation. Ferreira and Schmidt [51] found that individuals residing in slums in Rio de Janeiro, Brazil, were more susceptible to dengue infection. Additionally, by categorizing patients into mean income intervals in Cambé, Paraná, Brazil, the authors observed that dengue and income were negatively correlated, indicating that poorer areas were more likely to have a higher than average risk of dengue [46]. In another study from the ARBOALVO project, a stratification methodology using the Territorial Receptivity Index (TRI) for urban arboviruses was introduced, which served as a tool for the surveillance and control of dengue, Zika, and chikungunya. The authors analyzed various sociosanitary indicators strongly correlated with areas of greater susceptibility to urban arboviruses, which were directly or indirectly associated with conditions of poverty and lack of sanitation infrastructure [25].

Our results included the prediction of dengue case occurrences for each neighborhood, four weeks ahead, based on a single fit in the municipality of Natal. The best-fit model captured the spatial heterogeneity of the dengue case series as well as the peaks and nonoccurrence periods of the disease. The selected model enabled the generation of forecasts for each neighborhood, accurately predicting case peaks, periods of no occurrences, and projecting cases even under significantly different conditions, as observed in neighborhoods like Mãe Luíza, Pajuçara, Ponta Negra, and Potengi. The credibility intervals successfully captured the future values of reported dengue cases in a four-week horizon, except during moments of sharp and rapid growth. When updated at an appropriate frequency, the model quickly adapted to the accelerated growth in dengue cases, providing satisfactory forecasts. In all evaluated weeks, the model predicted high case counts in neighborhoods with elevated dengue occurrences. Additionally, the model accurately stratified areas in Natal, by identifying regions with low or no dengue occurrences.

Models with a space-time dependency structure for epidemiological counts commonly assume responses with a Poisson distribution. However, such models are inadequate in contexts where the observation of zero counts is frequent (a problem referred to in the literature as “excess of zeros”). To address this limitation, several studies have made use of hurdle and zero-inflated count models, such as the Hurdle Poisson and Zero-inflated Poisson models adopted in this work [53–56]. Better fits were obtained for periods and neighborhoods in Natal with null counts of dengue cases by incorporating a predictor structure based on entomological and sociosanitary indicators, as well as space-time effects. The structural flexibility of the model enabled it to capture not only the periods and neighborhoods without any occurrences, but also those with high dengue case counts. A limitation of the models adopted in this study is their inability to distinguish between true zeros (actual absence of cases) and zeros due to underreporting. Freitas et al. [57] addressed this issue by employing a Markov-switching model with latent states representing the presence or absence of cases. Furthermore, estimating the transition matrix between states of absence and presence accommodates long sequences of zeros, as well as prolonged sequences of positive counts interspersed with zeros, which may not be effectively handled by conventional zero-inflated structures.

We assumed the simplifying hypothesis of proportionality between spatiotemporal effects for the frequency and occurrence of dengue cases in Natal. More general relationships between these effects can be explored. For instance, in a multivariate spatial context, Schmidt et al. [58] employed distinct specifications for the spatial structures associated with dengue, Zika, and chikungunya in the city of Rio de Janeiro from 2015 to 2016.

From a predictive standpoint, it is noteworthy that our forecasts four steps ahead are conditioned on the lagged values of the entomological and climatic indicators. Therefore, the model relies on the previously observed values of the explanatory variables without relying on the establishment of hypothetical scenarios, except for the number of cases lagged by one week.

Finally, we emphasize that obtaining predictive maps of dengue cases serves as a tool for public health management services and epidemiological surveillance, enabling them to anticipate risk stratification within a city or metropolitan region, considering its heterogeneity. In this study, we used predictive maps to identify regions that subsequently exhibited high, moderate, low or zero dengue rates. Additionally, quantifying the uncertainty associated with all estimates and forecasts through probability intervals with intuitive and practical interpretations allows for a comprehensive evaluation of the precision of the estimates obtained. The ability to temporally describe forecasts that account for the individual conditions of each neighborhood stands out as a valuable tool for public health management, facilitating more closely targeted and effective interventions.

## Supporting information

S1 Table Population distribution by neighborhood and region, 2015 – 2018. * The population by neighborhood is a projection based on the average annual growth rate calculated from the 2000 and 2010 census data.
(DOCX)

S2 Table Epidemiological, entomological, climatic and sociosanitary indicators constructed at the neighborhood level in Natal. * Variables selected using the LASSO procedure.
(DOCX)
