# Supplementary material for: ARBOALVO: A Bayesian spatiotemporal learning and predictive model for dengue cases in the endemic Northeast city of Natal, Rio Grande do Norte, Brazil
Source: PLoS Negl Trop Dis. 2025 Apr 29;19(4):e0012984. doi: 10.1371/journal.pntd.0012984 (PMC12101852; doi:10.1371/journal.pntd.0012984)
Supplement: S1 Table — * The population by neighborhood is a projection based on the average annual growth rate calculated from the 2000 and 2010 census data. (DOCX) [file pntd.0012984.s001.docx]

**Table S1 – Population distribution by neighborhood and region, 2015 - 2018**

| Neighborhood | Region | Population* | | | |
| --- | --- | --- | --- | --- | --- |
|  |  | 2015 | 2016 | 2017 | 2018 |
| Alecrim | East | 27,231 | 26,930 | 26,626 | 28,705 |
| Areia Preta | East | 4,373 | 4,474 | 4,576 | 1,956 |
| Barro Vermelho | East | 10,871 | 11,031 | 11,193 | 10,087 |
| Cidade Alta | East | 7,297 | 7,333 | 7,368 | 7,123 |
| Lagoa Seca | East | 5,444 | 5,373 | 5,301 | 5,791 |
| Mãe Luíza | East | 14,515 | 14,425 | 14,333 | 14,959 |
| Petrópolis | East | 5,689 | 5,723 | 5,758 | 4,871 |
| Praia do Meio | East | 5,003 | 5,051 | 5,098 | 4,770 |
| Ribeira | East | 2,267 | 2,276 | 2,286 | 2,222 |
| Rocas | East | 10,423 | 10,417 | 10,410 | 10,452 |
| Santos Reis | East | 5,165 | 5,068 | 4,970 | 5,641 |
| Tirol | East | 16,693 | 16,804 | 16,916 | 16,148 |
| Igapó | North | 29,540 | 29,688 | 29,836 | 28,819 |
| Lagoa Azul | North | 65,680 | 66,576 | 67,481 | 61,289 |
| Nossa Senhora da Apresentação | North | 89,140 | 91,055 | 92,988 | 79,759 |
| Pajuçara | North | 64,436 | 65,746 | 67,068 | 58,021 |
| Potengi | North | 58,489 | 58,620 | 58,753 | 57,848 |
| Redinha | North | 18,699 | 19,122 | 19,548 | 16,630 |
| Salinas | North | 1,296 | 1,320 | 1,344 | 1,177 |
| Candelária | South | 23,888 | 24,193 | 24,501 | 22,391 |
| Capim Macio | South | 23,663 | 23,848 | 24,034 | 22,760 |
| Lagoa Nova | South | 38,305 | 38,465 | 38,628 | 37,518 |
| Neópolis | South | 22,636 | 22,671 | 22,706 | 22,465 |
| Nova Descoberta | South | 12,461 | 12,460 | 12,459 | 12,467 |
| Pitimbú | South | 24,703 | 24,804 | 24,906 | 24,209 |
| Ponta Negra | South | 25,117 | 25,207 | 25,296 | 24,681 |
| Bom Pastor | West | 18,321 | 18,341 | 18,361 | 18,224 |
| Cidade da Esperança | West | 19,001 | 18,929 | 18,856 | 19,356 |
| Cidade Nova | West | 18,407 | 18,562 | 18,717 | 17,651 |
| Dix-Sept Rosado | West | 15,507 | 15,469 | 15,432 | 15,689 |
| Filipe Camarão | West | 53,052 | 53,471 | 53,895 | 50,997 |
| Guarapés | West | 10,991 | 11,142 | 11,295 | 10,250 |
| Nordeste | West | 11,555 | 11,562 | 11,569 | 11,521 |
| Nossa Senhora de Nazaré | West | 16,343 | 16,385 | 16,428 | 16,136 |
| Planalto | West | 38,025 | 39,418 | 40,823 | 31,206 |
| Quintas | West | 26,416 | 26,220 | 26,022 | 27,375 |

*** The population by neighborhood is a projection based on the average annual growth rate calculated from the 2000 and 2010 census data.**
