# Supplementary material for: ARBOALVO: A Bayesian spatiotemporal learning and predictive model for dengue cases in the endemic Northeast city of Natal, Rio Grande do Norte, Brazil
Source: PLoS Negl Trop Dis. 2025 Apr 29;19(4):e0012984. doi: 10.1371/journal.pntd.0012984 (PMC12101852; doi:10.1371/journal.pntd.0012984)
Supplement: S2 Table — * Variables selected using the LASSO procedure. (DOCX) [file pntd.0012984.s002.docx]

**Table S2 - Epidemiological, entomological, climatic, and sociosanitary indicators constructed at the neighborhood level in Natal-RN.**

| **Indicator** | **Name** | **Indicator** | **Name** |
| --- | --- | --- | --- |
| **Epidemiological Dimension** | | Average number of people per household | EXP7 |
| Number of dengue cases | C-Dengue | Percentage of people under 14 and over 65 years old | EXP8 |
| **Entomological Dimension** | | Number of complaints about intermittency by neighborhood | EXP9 |
| Egg density index | IDO | Percentage of complaints about intermittency by neighborhood | EXP10 |
| Egg positivity index | IPO | Number of strategic points per neighborhood | EXP11 |
| **Climatic Dimension** | | Percentage of strategic points per neighborhood | EXP12 |
| Daytime temperature (in °C) | Temp-D | Number of complaints about intermittency divided by the number of households and multiplied by a thousand | EXP13 |
| Nighttime temperature (in °C) | Temp-N | Number of strategic points divided by the number of households and multiplied by a thousand | EXP14* |
| Rainfall (mm) | Chuva | Percentage of households with exclusive use bathrooms connected to the general sewage network or septic tank | VUL1 |
| **Socio-sanitary Dimension** | | Percentage of households with exclusive use bathrooms and sewage via rudimentary pit, trench, river, lake, sea, or other | VUL2* |
| Percentage of occupied area | Ocupada | Percentage of households without bathroom or exclusive use toilet for residents | VUL3 |
| Percentage of favela area | Fav | Percentage of households in irregular occupation situation (not owned, loaned, or rented) | VUL4 |
| Percentage of urban area increment from 1985 to 2000 | I-8500 | Percentage of households with irregular energy source | VUL5 |
| Percentage of urban area increment from 2000 to 2017 | I-0017 | Percentage of households with open sewage in the vicinity | VUL6* |
| Average altitude of neighborhoods (meters) | Altimetria | Percentage of households without public lighting in the vicinity | VUL7* |
| Percentage of vegetation | Vegetação | Percentage of households without pavement in the vicinity | VUL8 |
| Percentage of households connected to the general water supply network | EXP1 | Percentage of households without a manhole in the vicinity | VUL9 |
| Percentage of households with supply by well, rainwater stored in cistern, and other supply forms | EXP2* | Percentage of inadequate dwellings (without water, sewage, and garbage collection) | VUL10 |
| Percentage of households with collected garbage | EXP3 | Percentage of households with monthly per capita income up to one minimum wage | VUL11 |
| Percentage of households with garbage thrown in vacant lot, river, lagoon, sea, or other | EXP4* | Percentage of household heads under age | VUL12 |
| Percentage of households with accumulated garbage in the vicinity | EXP5* | Percentage of white population | VUL13* |
| Population density per occupied area | EXP6* | Density of poor per occupied area | VUL14* |

* Variables selected using the LASSO procedure.
